# Exosome-Integrated Hydrogels for Bone Tissue Engineering

**DOI:** 10.3390/gels10120762

**Published:** 2024-11-23

**Authors:** Hee Sook Hwang, Chung-Sung Lee

**Affiliations:** 1 Department of Pharmaceutical Engineering, Dankook University, Cheonan 31116, Republic of Korea; 2 Department of Pharmaceutical Engineering, Soonchunhyang University, Asan 31538, Republic of Korea

**Keywords:** exosome, hydrogel, nanocomposite, bone tissue engineering, regenerative medicine

## Abstract

Exosome-integrated hydrogels represent a promising frontier in bone tissue engineering, leveraging the unique biological properties of exosomes to enhance the regenerative capabilities of hydrogels. Exosomes, as naturally occurring extracellular vesicles, carry a diverse array of bioactive molecules that play critical roles in intercellular communication and tissue regeneration. When combined with hydrogels, these exosomes can be spatiotemporally delivered to target sites, offering a controlled and sustained release of therapeutic agents. This review aims to provide a comprehensive overview of the recent advancements in the development, engineering, and application of exosome-integrated hydrogels for bone tissue engineering, highlighting their potential to overcome current challenges in tissue regeneration. Furthermore, the review explores the mechanistic pathways by which exosomes embedded within hydrogels facilitate bone repair, encompassing the regulation of inflammatory pathways, enhancement of angiogenic processes, and induction of osteogenic differentiation. Finally, the review addresses the existing challenges, such as scalability, reproducibility, and regulatory considerations, while also suggesting future directions for research in this rapidly evolving field. Thus, we hope this review contributes to advancing the development of next-generation biomaterials that synergistically integrate exosome and hydrogel technologies, thereby enhancing the efficacy of bone tissue regeneration.

## 1. Introduction

Bone tissue engineering represents a significant advancement in regenerative medicine, aimed at developing innovative strategies to restore the function of damaged or diseased bone tissue [1,2]. Traditional bone repair techniques, including the use of autografts and allografts, are limited by several challenges such as donor site morbidity, limited graft availability, and the risk of immune rejection [3,4]. As a result, there has been a growing interest in tissue engineering approaches that can overcome these obstacles and provide more effective solutions for bone regeneration.

In this context, hydrogels have gained widespread attention in the field of tissue engineering due to their unique properties, such as high water content, biocompatibility, and the ability to mimic the extracellular matrix (ECM) of natural tissues [5]. These characteristics make hydrogels particularly well-suited for supporting cell growth and promoting tissue regeneration [6]. However, conventional hydrogel systems often lack sufficient bioactivity for effective bone regeneration. To address this, researchers are exploring innovative strategies to enhance their functional properties and improve their regenerative potential [7,8].

Exosomes, which are nano-sized extracellular vesicles released by cells, have emerged as a potent tool in tissue engineering due to their ability to mediate intercellular communication and modulate various biological processes, influencing various biological processes such as immune response, inflammation, and tissue repair [5,9]. Originating from the endosomal compartment of cells, these vesicles are rich in proteins, lipids, and nucleic acids, making them potent mediators of intercellular signaling [10,11]. Exosomes, measuring approximately 40–100 nm, are one of several subtypes of extracellular vesicles, distinguished by their phospholipid bilayer with endogenous surface markers and unique biogenesis pathway [12,13]. Exosomes are often categorized by the type of cells from which they originate. Additionally, exosomes are secreted by most cell types and can be found in various body fluids, with their content influenced by the cell of origin and its physiological state [14,15]. Each type of exosome carries unique surface markers and cargo reflective of its cell of origin, which can influence its therapeutic potential and target specificity [16,17]. These characteristics make exosomes particularly advantageous for applications in regenerative medicine, where precise and sustained delivery of therapeutic agents is crucial. Diverse cargo of exosomes includes DNA, mRNA, microRNA, long non-coding RNA, and proteins, which are essential for their role in regulating cellular pathways and signaling processes [18,19]. Furthermore, integrating exosomes into hydrogel matrices significantly boosts the bioactivity of these scaffolds, enabling a controlled and sustained release of bioactive molecules that support tissue regeneration [9,20]. This integration offers a promising approach to addressing the limitations of conventional hydrogels by improving their mechanical strength and biological functionality.

**Figure 1:**
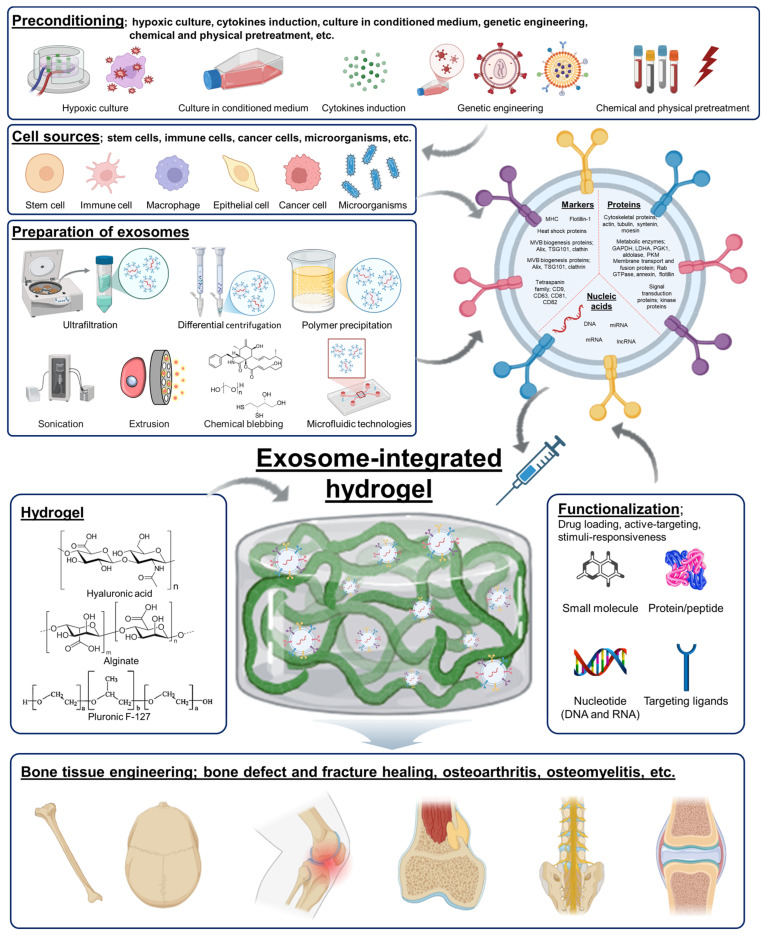
Exosome-integrated hydrogels for bone tissue engineering. Exosomes, which develop from the endosomal system within cells, are packed with proteins, lipids, and nucleic acids that facilitate robust intercellular communication. Exosomes, typically around 40–100 nm in size, belong to a diverse group of extracellular vesicles, set apart by their phospholipid membrane and specific formation pathway. They are released by a variety of cell types and circulate in bodily fluids, with their molecular composition reflecting the origin and condition of the parent cell. Exosomes can be isolated and fabricated through differential centrifugation, density gradient centrifugation, ultrafiltration, size exclusion chromatography, affinity nanoparticle-based isolation, polymer precipitation, microfluidic technologies, etc. Exosomes contain diverse bioactive molecules such as proteins, nucleic acids, and lipids, with two main types of proteins: general markers (like CD9, CD63, and CD81) and proteins unique to their parent cell. Exosomes can be modified with drug loading, active targeting, and stimuli-responsiveness. Hydrogels have become valuable tools in biomedical applications for exosome delivery. Hydrogels, as water-retentive polymer networks, encapsulate exosomes, improving their retention at target sites and providing controlled release to enhance localized effects. These exosome–hydrogel composites show promise in areas such as bone tissue engineering and treatment. (Created with https://biorender.com).

The combination of exosomes with hydrogels thus opens new avenues for the development of advanced biomaterials tailored for bone tissue engineering [21,22]. This review will explore the recent advancements in the development, engineering, and application of exosome-integrated hydrogels, highlighting their transformative potential in bone tissue engineering (Figure 1). The following sections explore the development, engineering, application, and mechanistic pathways of exosome-integrated hydrogels, while also discussing challenges like scalability and regulatory issues, and providing future research directions.

## 2. Exosomes in Bone Tissue Engineering

In the context of bone tissue engineering, exosomes have garnered significant attention due to their ability to modulate the behavior of target cells, thereby promoting bone regeneration and repair [23,24]. Their specific influence on cellular activities, such as migration, proliferation, collagen synthesis, and angiogenesis, underscores their potential in tissue repair and regenerative medicine. The following section provides a detailed discussion on the role of exosomes in promoting bone regeneration. Here, we emphasize the key components of exosomes and their respective functions. The primary focus of this review is on the mechanisms by which exosomes facilitate bone regeneration. Thus, we outline the regenerative process and the factors that influence it, followed by an in-depth analysis of how exosomes contribute to the repair of bone defects. We utilized Google Scholar as our primary database. Our inclusion criteria focused primarily on studies published within the last five years. In addition, we also included some key studies published within the last ten years to provide a comprehensive understanding of foundational and significant advancements in this area.

### 2.1. Preparation and Characteristics of Exosomes

Exosomes can originate from various sources, including mammalian cells (e.g., immune cells, stem cells, and tumor cells), plants, and microorganisms like bacteria and fungi [25]. This wide range of sources provides exosomes with diverse cargo profiles and functionalities, making them versatile tools for biomedical research and therapeutic applications.

Exosomes from mammalian cells, such as macrophages, dendritic cells (DCs), B cells, T cells, platelets, epithelial cells, reticulocytes, mast cells, neurons, oligodendrocytes, tumor cells, and Schwann cells, are of significant interest in clinical settings [17]. Mammalian cell-derived exosomes are found to have similar functions to their originating cells but with lower immunogenicity [26]. As such, they are being explored for their roles in bone tissue engineering, chronic wound healing, kidney diseases, and certain cancers [27,28,29]. These exosomes can also be harvested from biological fluids such as plasma, urine, saliva, semen, breast milk, cerebrospinal fluid, and bile [30,31]. Plant-based sources, such as ginger, garlic, grapefruit, carrots, and broccoli, have been explored for their exosome content, especially due to their anti-inflammatory properties [32,33,34,35,36]. These plant exosomes can carry therapeutic agents, as seen in ginger-derived nanocarriers with antitumor properties and ginseng-based exosomes capable of crossing the blood–brain barrier [34,37,38]. Fruits like strawberries, blueberries, grapes, apples, and lemons are also considered scalable, safe sources of exosomes [25,33]. Additionally, exosomes from mammalian milk (e.g., bovine, porcine, buffalo, and human breast milk) are being investigated for therapeutic applications [39,40,41,42].

In recent years, microbe-derived exosomes have gained attention for use in drug delivery systems and other biomedical applications [43,44]. Bacterial extracellular vesicles (BEVs) are capable of delivering genetic material and proteins, influencing processes like immune response, homeostasis, and cell death [45,46,47,48].

Exosomes derived from mesenchymal stem cells (MSCs) have garnered particular interest in regenerative medicine [5,49]. These vesicles, obtained from different MSC sources such as adipose tissue, bone marrow, and umbilical cord, exhibit specific interactions with osteoblasts and osteoclasts, both crucial in bone tissue repair [50]. The variability of exosomes underscores the significance of the source cell, as well as the cell culture conditions (cell density, passage number, and culture time), which together impact the exosome’s regenerative capabilities [26,51]. Furthermore, bone marrow-derived mesenchymal stem cells (BM-MSCs), adipose-derived MSCs (AD-MSCs), and umbilical cord-derived MSCs (UC-MSCs) have been extensively investigated in the context of stem cell-derived exosome therapies, suggesting that these stem cell types may serve as superior sources for exosome isolation [5]. Notably, the amount of exosomes obtained from human BM-MSCs has been reported to be lower compared to those derived from human amniotic fluid MSCs. Additionally, research conducted by Tracy et al. revealed that the total exosome yield, when adjusted for the number of cells, was significantly greater in amniotic fluid MSCs compared to BM-MSCs [52].

Exosome biogenesis begins within the endosomal system and involves a series of complex steps [15,53,54,55,56]. First, proteins, nucleic acids, and lipids are synthesized in the cytoplasm, providing the foundation for exosome formation. Second, these components travel through the endoplasmic reticulum (ER) and Golgi apparatus for further modification and packaging into multivesicular bodies (MVBs). Third, within the MVBs, inward budding of the membrane encapsulates these molecules, forming intraluminal vesicles. Fourth, some of these vesicles are retained within the MVBs, eventually leading to the formation of larger exosomes. Finally, through the process of exocytosis, exosomes are released into the extracellular space when MVBs fuse with the plasma membrane.

Efficient exosome isolation is critical for cell-free exosome therapies. Among the various isolation techniques, differential ultracentrifugation is often regarded as the “gold standard”, but it requires expensive equipment and long processing times (Table 1) [57]. The high centrifugal forces used in differential ultracentrifugation may also damage exosome membranes, affecting their functional analysis. Density gradient ultracentrifugation, an enhanced version of differential ultracentrifugation, separates exosomes using media of varying densities, such as sucrose and iodixanol, achieving higher purity but with complex preparation steps, limiting its scalability [58,59]. Ultrafiltration is a simpler, size-based technique that allows rapid processing of large samples but can suffer from membrane clogging, raising costs [60,61,62,63]. Size exclusion chromatography separates exosomes based on molecular size and yields purer samples, though it may introduce nonspecific protein contamination [64]. Polymer precipitation, using agents like polyethylene glycol (PEG), aggregates exosomes by altering ionic strength [62,65]. However, this method can introduce contaminants from organic solvents. In addition, affinity nanoparticle-based methods, which employ ligands that specifically bind exosome surface markers, offer high selectivity and are effective for rapid exosome isolation [66,67,68]. While these methods preserve exosome integrity, the high costs of commercial kits limit their large-scale application. Lastly, microfluidic technologies offer several advantages for exosome isolation, including automated processing, which increases production efficiency by minimizing manual intervention [69]. These systems allow for high-throughput and label-free separation of exosomes based on their physical or biochemical properties, enhancing precision in exosome detection and isolation [70,71]. Additionally, microfluidic platforms can target exosome-specific surface proteins, providing higher specificity than traditional size-based methods [72,73,74]. However, the high cost of the required equipment and the need for operators with specialized skills pose significant challenges. This makes the widespread application of microfluidic methods more limited in resource-constrained settings.

Exosomes are known to carry specific marker proteins, such as CD9, CD63, CD81, and heat shock proteins (HSP27, HSP70, HSP90), along with integrins and cell adhesion molecules (CAMs) [56,75]. These surface markers facilitate prolonged circulation and enhanced stability in the body, adding to their therapeutic appeal. In addition, these proteins, including TSG101, can serve as valuable diagnostic biomarkers in certain diseases. To accurately identify and quantify these markers, various techniques such as enzyme-linked immunosorbent assays (ELISA), SDS-polyacrylamide gel electrophoresis (SDS-PAGE), bicinchoninic acid (BCA) assays, and Bradford assays are commonly used [76]. Western blotting and flow cytometry are also employed for the precise quantification of exosomal markers, while mass spectrometry, particularly MALDI-MS, has been utilized to investigate exosome biomarkers [77,78].

Exosomes also contain endogenous nucleic acids such as mRNA, miRNA, and siRNA, which are shielded from enzymatic degradation [79]. Due to their natural biocompatibility and low immunogenicity, exosomes have garnered significant interest for clinical applications, especially in cancer screening/early detection and targeted therapies [80]. Techniques like transmission electron microscopy (TEM) and nanoparticle tracking analysis (NTA) are used to visualize exosome structure and determine particle size [81,82]. The slight negative surface charge of exosomes, measurable by zeta potential analysis, contributes to their extended circulation time [83].

While exosomes hold promise, challenges such as limited production yield and storage stability have spurred the development of hybrid exosomes [5,84,85,86]. By combining the natural properties of exosomes with the structural advantages of synthetic nanoparticles, hybrid exosomes provide improved colloidal stability, extended half-life, and the ability to carry higher therapeutic payloads [84,87]. These hybrid structures are engineered through various methods, including extrusion, filtration, microfluidic devices, nitrogen cavitation, sonication, cell blebbing, freeze–thawing, and biohybrid approaches, to create advanced drug delivery systems [88,89]. Liposome-exosome fusion strategies have emerged to merge the endogenous biological activity of exosomes with the surface modification flexibility of liposomes, thus overcoming some limitations associated with each individual system [85,90].

As the field of exosome research advances, the potential for employing hybrid exosomes as drug delivery platforms in regenerative medicine also expands, providing enhanced target-specific delivery and improved biological stability [86]. By exploring different isolation techniques, optimizing culture conditions, and leveraging hybridization strategies, exosomes can be further refined for therapeutic applications, particularly in bone tissue engineering.

### 2.2. Role of Exosomes in Bone Tissue Engineering

Exosomes play a crucial role in bone tissue engineering due to their ability to regulate fundamental processes involved in bone regeneration, such as osteogenesis, angiogenesis, and immunomodulation [29]. By transferring their cargo to target cells, exosomes influence gene expression and cellular behaviors essential for effective bone repair [5]. Bone regeneration occurs through two primary processes: intramembranous and endochondral ossification [91,92,93]. Intramembranous ossification forms flat bones directly from mesenchymal cells, whereas endochondral ossification, responsible for long bone formation, uses cartilage as a template. These processes involve transcription factors like runt-related transcription factor 2 (RUNX2) and Osterix that promote osteoblast differentiation, with vascularization and angiogenic factors like vascular endothelial growth factor (VEGF) playing key roles in bone matrix formation and remodeling [94]. Exosomes have emerged as an important means of delivering osteogenic signals, enhancing bone regeneration by mediating intercellular communication more effectively than cytokine signaling, which has limitations due to its short half-life and limited range [95].

Exosomes derived from MSCs enhance osteogenesis by delivering bioactive molecules that promote the osteogenic differentiation of precursor cells [96,97,98]. These vesicles are loaded with osteogenic factors, including bone morphogenetic proteins (BMPs) and specific miRNAs (e.g., miR-27a-3p and miR-29a), which regulate genes like RUNX2, Osterix, and alkaline phosphatase (ALP) [51,94]. Exosomes also activate signaling pathways such as the Wnt/β-catenin pathway, which is crucial for bone formation and mineralization [99,100]. Moreover, mouse myoblasts (C2C12) and human-induced pluripotent stem cell-derived MSC-derived exosomes can modulate bone matrix mineralization by increasing calcium and phosphorus deposits in mouse preosteoblasts (MC3T3-E1) and bone marrow MSCs derived from ovariectomized (OVX) rats, as evidenced by elevated ALP activity and mineralized matrix formation in vitro and healing of critical size bone defects in ovariectomized rats [101,102].

Angiogenesis, or the formation of new blood vessels, is essential for delivering nutrients and oxygen to regenerating tissues [103,104]. Exosomes support angiogenesis by delivering pro-angiogenic factors such as VEGF and miRNAs (e.g., miRNA-210, miRNA-23a-3p, miRNA-424, miRNA-30b, miRNA-30c, and miRNA-126) to endothelial cells [105,106,107]. These molecules encourage endothelial cell proliferation, migration, and tube formation, enhancing the vascularization needed for successful bone graft integration [108]. A study has shown that human umbilical cord mesenchymal stem cells (UMSCs) exosomes can increase the expression of VEGF, hypoxia-inducible factor-1α (HIF-1α), further promoting vascularization, which is essential in areas with poor blood supply where bone regeneration might otherwise fail [109].

The immune response is a double-edged sword in bone regeneration: an initial inflammatory response is necessary for initiating repair, but prolonged inflammation can hinder healing [110,111,112]. Exosomes exhibit immunomodulatory properties by influencing immune cell polarization to favor bone regeneration [98,113,114]. Particularly, MSC-derived exosomes can suppress pro-inflammatory macrophages (M1 phenotype) and promote anti-inflammatory macrophages (M2 phenotype) [115]. By inducing M2 macrophage polarization, human bone marrow MSC-derived exosomes overexpressing miRNA-181b contribute to reduced inflammation and foster a pro-healing environment, essential for the success of bone tissue engineering [116,117]. Certain genetic material (mi-RNA-181b) carried by exosomes can facilitate M2 macrophage polarization by suppressing the expression of protein Kinase C Delta (PRKCD) and phosphatase and tensin homologue (PTEN), thereby enhancing p-AKT expression and activating the phosphoinositide 3-kinase/protein kinase B (PI3K/AKT) signaling pathway [117]. Additionally, exosomal proteins have been shown to play a role in macrophage polarization. Research by Nakao et al. and colleagues demonstrated that human gingival tissue-derived MSC-derived exosomes stimulated by tumor necrosis factor-α (TNF-α) could promote M2 polarization of macrophages derived from human CD14+ peripheral blood-derived monocytes by increasing CD73 expression [118]. Moreover, the exosomes can modulate the function of T cells and dendritic cells, creating an immune environment supportive of tissue regeneration [50]. Integrating exosomes with bone tissue engineering scaffolds not only enhances the osteogenic potential of exosomes but also mitigates the excessive inflammatory response commonly triggered by scaffold implantation, thus promoting improved bone regeneration [29,119].

Exosomes’ unique function as mediators of intercellular communication makes them a valuable tool for bone tissue engineering. They not only promote osteogenic differentiation and enhance angiogenesis but also regulate immune responses, thereby creating an optimal environment for bone healing. As research progresses, exosomes could potentially be combined with tissue-engineered scaffolds to maximize their regenerative potential while minimizing the inflammatory response associated with scaffold implantation.

### 2.3. Advanced Engineering of Exosomes

Recently, researchers have increasingly focused on engineering exosomes to enhance their therapeutic efficacy and optimize their functionality for clinical applications (Table 2). Advanced engineering of exosomes can be broadly categorized into several key approaches. Modifying the membrane proteins on exosome surfaces can enable specific targeting and improve their binding affinity for target cells, which enhances the selective uptake by tissues or cell types. Emani et al. reported that exosomes derived from cardiosphere-derived cells, modified with targeted cell membrane peptides, showed a 3.8-fold increase in uptake by primary cardiomyocytes compared to non-engineered controls, highlighting the potential for specific tissue targeting [120]. In another study, Zhu et al. employed embryonic stem cells (ESCs) to produce exosomes for use as a tumor-targeting delivery platform [121]. To enhance targeting capability, they functionalized the surface of these exosomes with the c(RGDyK) peptide, which binds selectively to tumor tissues such as glioblastoma, prostate, and lung cancers. This was achieved through post-lipid incorporation of DSPE-PEG-c(RGDyK). The modified exosomes were shown to efficiently deliver the chemotherapeutic drug paclitaxel to glioblastoma sites, demonstrating up to an eightfold reduction in tumor volume in a glioblastoma mouse model, as observed with U87 and U251 cell lines. Particularly, modifying exosome surfaces with antigens or antibodies allows them to induce immune responses or enhance immune system interactions [122]. Exosomes engineered with single-chain MHCI trimers on antigen-presenting surfaces have been found to amplify tumor-specific CD8+ T cells, effectively triggering anti-tumor immune responses. This highlights the potential for exosomes in immunotherapy applications. Next, altering the lipid composition of exosomes can improve their stability, biodistribution, and biological activity under physiological conditions. This can be achieved by modulating the lipid metabolism of source cells or through post-production modifications [5,84]. Jin et al. reported that lipopolysaccharide-stimulated macrophages can increase the abundance of phosphatidylinositol-4-phosphate (PI4P) on multivesicular bodies, promoting the release of small extracellular vesicles [123]. Such modifications enhance the stability and functionality of exosomes within physiological environments.

Additionally, loading exosomes with therapeutic agents, including drugs, nucleic acids, and proteins, can transform them into effective drug delivery vehicles. This can be achieved by altering the gene expression of source cells to naturally incorporate specific cargo or by directly modifying the exosome membrane. Zhu et al. successfully loaded the anti-proliferative drug doxorubicin onto amine-modified intraocular lenses electrostatically, significantly inhibiting the development of posterior capsule opacification [124]. In another study, exosome mimetic vesicles loaded with Smoothened agonists by the extrusion process enhanced the osteogenic capacity through the upregulation of hedgehog signaling [10]. These methods emphasize the versatility of exosomes in delivering bioactive compounds to specific sites in the body. Additionally, modifying exosomes to carry specific miRNAs or mRNAs allows them to regulate gene expression in target cells [51,125]. Fan et al. produced exosome mimetics using an extrusion method from human mesenchymal stem cells (hMSCs), yielding a higher proportion of vesicles positive for exosome-specific markers [51]. Genetic modification of hMSCs, specifically down-regulating noggin expression, enhanced the osteogenic properties of exosomes, leading to improved bone regeneration through modulation of miR-29a signaling. Moreover, engineered exosomes containing miR-31 have been shown to promote angiogenesis, fibrogenesis, and re-epithelialization by inhibiting factors such as epithelial membrane protein 1 (EMP-1) and HIF-1AN (also known as FIH) [125]. This approach shows the potential of exosome-based gene therapies for enhancing tissue repair processes.

Despite these advancements, challenges remain in terms of exosomes’ biological distribution, metabolic stability, and retention within the body. Due to their small size, exosomes are vulnerable to rapid filtration by the kidneys, leading to excretion and reduced circulation time [126,127]. Additionally, they may be recognized and cleared by macrophages in the immune system, particularly when entering the bloodstream [128]. Factors such as pH changes, ionic strength variations, and enzymatic degradation by proteases and nucleases further reduce exosome stability and functionality in vivo [129,130]. To mitigate these challenges, PEG (polyethylene glycol) modification has emerged as a promising strategy. PEGylation extends exosome circulation time, reduces immune clearance, and enhances their stability under physiological conditions [131,132,133]. By selecting PEG molecules with functional groups that form stable covalent bonds with amino groups on exosome surfaces, PEG chains can be anchored to the vesicle’s lipid bilayer, increasing both stability and longevity. Another approach is to use lipophilic PEG variants, such as DSPE-PEG, which embed in the lipid bilayer and extend outward, providing a protective barrier [134]. Furthermore, incorporating PEGylation during exosome production can yield PEG-modified exosomes directly from the source cells, optimizing the process for scalable production.

By employing these engineering strategies, exosomes can be fine-tuned to meet the specific needs of clinical applications. Whether through surface modification for targeted delivery, cargo engineering for therapeutic payloads, or PEGylation for enhanced stability, advanced exosome engineering offers promising avenues for developing effective next-generation therapies in bone tissue engineering and beyond.

**Table 2:** Key approaches for engineered exosomes.

Engineering Approach	Materials	Description	Example	Ref.
Surface protein modification	Embryonic stem cell-derived exosomes modified with c(RGDyK) and paclitaxel loading	Modifying exosome surface membrane proteins to enhance specific targeting and/or affinity for target cells, thereby improving uptake efficiency.	Embryonic stem cell-derived exosomes for tumor-targeting delivery through surface functionalization with the c(RGDyK) peptide via postlipid incorporation of DSPE-PEG-c(RGDyK), demonstrating up to an eightfold reduction in tumor volume.	[121]
	Exosomes isolated from adipose-derived stem cells with dextran sulfate displayed on the surface of the exosomes		Dextran sulfate as a targeting moiety was introduced onto the surface of exosomes through metabolic glycoengineering and click chemistry, demonstrating enhanced therapeutic efficacy at a concentration ten times lower for rheumatoid arthritis treatment.	[135]
Surface engineering with antigens/antibodies	Exosomes derived from HEK293 cells stably expressing ovalbumin peptide-single chain trimer-CD81-IL2 and CD80-CD9	Adding antigens or antibodies to exosome surfaces to induce immune responses or enhance immune interactions.	Antigen-presenting exosomes modified with single-chain MHCI trimer amplified tumor-specific CD8+ T cells, inducing anti-tumor effects.	[122]
Lipid composition modification	Macrophage-derived extracellular vehicles	Altering lipid composition of extracellular vehicles including exosomes to improve stability, biodistribution, and biological activity in vivo.	LPS-stimulated macrophages increased phosphatidylinositol-4-phosphate (PI4P) levels, enhancing small extracellular vesicle release.	[123]
Cargo engineering	Human lens epithelial cell-derived exosomes with doxorubicin loading and foldable hydrophobic acrylic intraocular lens	Loading exosomes with drugs, nucleic acids, or proteins transforms them into drug delivery vehicles.	Anti-proliferative drug doxorubicin loaded onto amine-modified intraocular lenses, significantly inhibiting posterior capsule opacification.	[124]
	Human bone mesenchymal marrow stem cell-derived exosomes modified with alendronate and Smoothened agonist (SAG) loading/hydroxyapatite-coated PLGA scaffold		Exosomes loaded with Smoothened agonists by extrusion process improve osteogenic capacity by activating hedgehog signaling.	[10]
miRNA/mRNA engineering	Genetically engineered HEK293 cell-derived exosomes with miR-31-5p lentiviral vector system	Modifying exosomes to carry specific miRNA or mRNA allows regulation of gene expression in target cells.	Exosomes engineered with miR-31 promote angiogenesis, fibrogenesis, and re-epithelialization by inhibiting EMP-1 and FIH.	[125]
	Genetically engineered human bone marrow mesenchymal stem cell-derived exosomes with noggin lentiviral vector system		Genetic modification of hMSCs, specifically down-regulating noggin expression, enhanced the osteogenic properties of exosomes, leading to improved bone regeneration through modulation of miR-29a signaling.	[51]
PEGylation	Rat mesenchymal stem cell-derived exosomes modified with DSPE-PEG * through lipid insertion into the phospholipid bilayer/gelatin methacryloyl (GelMa) hydrogel	PEG modification to improve exosome stability, circulation time, and reduce immune clearance in the body.	PEG covalently attaches to lysine residues on exosomes, while DSPE-PEG inserts into the lipid bilayer, extending the PEG chain outward.	[131]

* 1,2-Dioleoyl-sn-glycero-3-phosphoetha-nolamine-poly(ethylene glycol).

### 2.4. Challenges and Opportunities in Exosome-Based Bone Tissue Engineering

While exosomes hold great promise for bone tissue engineering, there are several challenges that need to be addressed to fully realize their potential. One of the primary challenges is the isolation and purification of exosomes, which is a labor-intensive and time-consuming process. The heterogeneity of exosomes also poses a challenge, as the therapeutic efficacy can vary depending on the source and condition of the parent cells. Moreover, the scalability of exosome production is a critical issue that needs to be addressed for clinical applications.

Despite these challenges, the opportunities for exosome-based therapies in bone tissue engineering are vast. Advances in biomanufacturing technologies, such as bioreactors and microfluidic devices, are being explored to enhance the production and purity of exosomes. Additionally, the development of standardized protocols for exosome isolation and characterization will be crucial for ensuring the reproducibility and efficacy of exosome-based therapies.

Furthermore, the integration of exosomes into biomaterials, such as hydrogels, opens new avenues for the development of advanced scaffolds that can mimic the natural bone environment. These exosome-laden hydrogels can provide sustained and localized delivery of exosomes to the defect site, thereby enhancing the regenerative potential of the scaffold. The combination of exosomes with other bioactive molecules or cells can also be explored to create multifunctional scaffolds that address the complex requirements of bone regeneration.

In conclusion, exosomes represent a powerful tool in bone tissue engineering, offering a cell-free approach to enhance bone regeneration through the delivery of bioactive molecules that modulate osteogenesis, angiogenesis, and immunomodulation. While there are challenges to be overcome, the continued research and development in this field hold great promise for the future of regenerative medicine and the treatment of bone defects.

## 3. Exosome-Integrated Hydrogels

Since the 1980s, scholarly interest in hydrogels and exosomes has grown significantly, with research focusing on hydrogels’ hydrophilic nature and unique ability to retain large amounts of water without dissolving [3,136,137,138,139]. This high water content allows hydrogels to absorb between 10% and several thousand times their dry weight in water, depending on the degree of cross-linking in the polymer network. The cross-linked structure provides structural integrity and facilitates nutrient and waste exchange, essential for cell viability and function, thus mimicking the natural ECM [25,140].

Hydrogels have become a cornerstone in tissue engineering due to their ability to closely mimic the extracellular matrix (ECM) of natural tissues [140]. Their highly hydrated, three-dimensional network structure, biocompatibility, and customizable mechanical properties make them ideal for creating scaffolds that support tissue regeneration [141,142,143]. In bone tissue engineering, hydrogels serve as both a structural scaffold and a delivery system for bioactive agents, such as exosomes, growth factors, and cells [5]. This dual role provides a conducive environment for cell growth while enabling the controlled release of therapeutic agents, enhancing their overall utility. The incorporation of bioactive agents into hydrogels can be achieved through various methods, including physical entrapment, chemical conjugation, or embedding during hydrogel formation [144,145]. Physical entrapment involves dispersing the bioactive agents within the hydrogel matrix during gelation, while chemical conjugation binds the agents covalently to the polymer network, ensuring sustained release. This flexibility enables hydrogels to release encapsulated agents, such as exosomes, in a controlled manner, with release profiles tailored for either rapid delivery or sustained release [21,146]. This feature is essential for bone tissue engineering, where timely delivery of growth factors, exosomes, and other therapeutic agents is critical for successful tissue regeneration. Moreover, traditional delivery methods, such as intravenous or subcutaneous injection, often lead to exosome accumulation in non-target organs, such as the liver and spleen, due to rapid systemic clearance. In contrast, embedding exosomes within hydrogels allows localized delivery, prolonging retention and improving therapeutic efficacy. Hydrogels with passive hydrolytic or cell-mediated enzymatic degradation can further optimize exosome release profiles, supporting a controlled and sustained delivery of therapeutic agents.

Hydrogels can be composed of either natural or synthetic polymers, or a combination of both, allowing for extensive customization of their properties [147,148,149]. Natural hydrogels, such as protein hydrogels (e.g., collagen), polysaccharides (e.g., hyaluronic acid, alginate), and DNA hydrogels, are favored for their biocompatibility and bioactivity, which support cellular functions and tissue regeneration [3,25]. Synthetic hydrogels, including polyvinyl alcohol and acrylate-based hydrogels, offer greater control over mechanical properties, degradation rates, and functionalization, making them suitable for specific applications in bone tissue engineering [150]. These properties can be further tailored through chemical or physical cross-linking, which allows hydrogels to respond to environmental changes, such as pH, temperature, or biomolecule concentration, enhancing their versatility for therapeutic applications [151,152,153,154]. Additionally, one of the key advantages of hydrogels is their tunable mechanical properties. By adjusting the crosslinking density, polymer concentration, and type of polymers used, hydrogels can range from soft, gel-like structures suited for soft tissue engineering to rigid structures capable of supporting bone regeneration [155]. Furthermore, incorporating nanomaterials within exosome-integrated hydrogels may offer another avenue for enhancing bone tissue engineering applications [136,156]. Nanomaterials can potentially improve the mechanical properties and bioactivity of hydrogels, facilitating better integration with bone tissue. For bone tissue engineering, it is crucial that hydrogels have sufficient mechanical strength to withstand physiological loads while maintaining a porous structure that allows for cell infiltration and vascularization.

Encapsulation of exosomes within hydrogels can be achieved using several techniques, each tailored to the desired release kinetics and application. In situ gelation involves mixing exosomes with polymers and crosslinkers, followed by injection at the target site, where gelation occurs due to environmental triggers such as irradiation or pH changes [157,158,159]. Xiao et al. studied an injectable temperature-responsive hydrogel with exosomes to control inflammation and oxidative damage in acute spinal cord injury [160]. A PLGA-PEG-PLGA hydrogel was used for thermo-responsive in situ gelation for delivery and localization of exosomes after injection. Furthermore, Fan et al. reported local exosome delivery with methacrylated chitosan for bone regeneration. This modified chitosan polymer was integrated with exosomes using riboflavin as a catalyst, injected into the defect site, and then photocrosslinked [51]. This method allows the hydrogel to adapt to irregular cavities, ensuring high retention and integration. Pre-mixed gelation involves combining exosomes with polymers prior to adding crosslinkers for gelation. A typical example is a composite matrix of thiolated hyaluronic acid, heparin, and gelatin, crosslinked with PEG diacrylate (PEGDA), which offers both structural stability and improved exosome retention and release [161]. Optimizing reaction conditions like temperature and using non-toxic crosslinkers such as genipin helps reduce cytotoxicity from any unreacted crosslinkers. Post-gelation loading involves pre-forming the hydrogel via polymerization and then soaking it in an exosome solution [26,140]. The exosomes are absorbed into the hydrogel’s porous structure through its swelling capacity. However, pore size must be carefully controlled: pores that are too large may lead to exosome leakage, while pores that are too small may hinder exosome entry. By applying these various encapsulation techniques, exosome-integrated hydrogels can be customized to offer sustained and localized delivery of therapeutic agents in bone tissue engineering, effectively boosting the regenerative potential of exosomes while providing a supportive structure for cell growth and interaction.

Exosome-integrated hydrogels represent an innovative approach in bone tissue engineering, combining the therapeutic potential of exosomes with the structural and mechanical benefits of hydrogels [140,146]. By embedding exosomes within hydrogels, premature clearance of these extracellular vesicles is minimized, providing sustained, localized delivery to target sites. The cross-linked hydrogel matrix supports a concentrated dose of exosomes at the application site, resulting in enhanced therapeutic effects. Together, the exosome’s cell-signaling capabilities and the hydrogel’s customizable properties create a platform that enhances bone regeneration, representing a promising avenue for advanced tissue engineering strategies.

## 4. Recent Innovations and Applications in Bone Tissue Engineering

Exosome-integrated hydrogels are emerging as a transformative solution in bone tissue engineering, leveraging the biological activity of exosomes and the structural properties of hydrogels to improve bone regeneration outcomes. By combining these two components, researchers aim to mimic the natural ECM, enhancing cell proliferation, migration, and differentiation, which are critical for effective bone repair. This section explores recent advancements in exosome-integrated hydrogels for addressing various bone-related conditions (Table 3).

### 4.1. Bone Defect Healing

Bone defects resulting from trauma, infection, tumors, congenital anomalies, and other conditions have posed significant challenges in clinical settings [181]. Traditionally, autologous and allogeneic bone grafts have been widely employed for bone repair, but their limitations, such as donor site morbidity and immune rejection, have led to the exploration of alternative strategies [6,182,183,184].

The use of hydrogel-exosome systems represents an innovative therapeutic approach due to their adaptability and potential to enhance bone regeneration. The exosomes used in these systems can be preconditioned through various techniques to improve their regenerative potential [51,163,185,186]. Mesenchymal stem cells can be immunologically trained using lipopolysaccharide (LPS) to enhance their immunomodulatory effects [187,188]. The exosomes derived from LPS-stimulated MSCs inherit the enhanced reparative capabilities, particularly in promoting M2 macrophage polarization via the inhibition of PKM2 translocation into the nuclei of pro-inflammatory macrophages [162]. Injectable silk fibroin hydrogels with osteogenic nanoclay, Laponite, are used to deliver these immunomodulatory exosomes, showing controlled gelation and promoting healing in alveolar-bone defects in rats.

**Figure 2:**
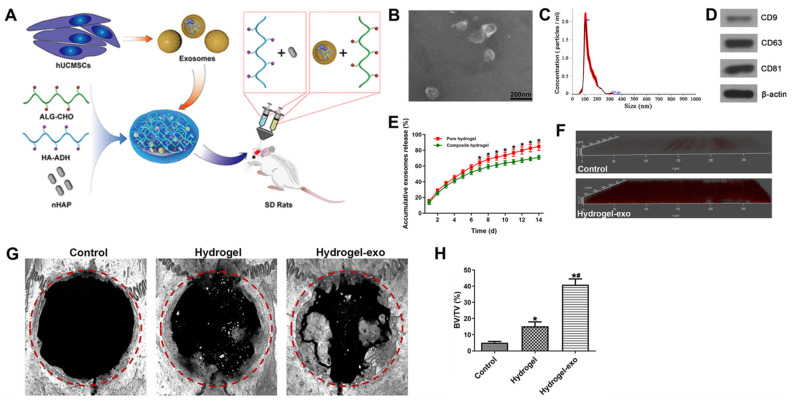
Human umbilical cord mesenchymal stem cell-derived exosomes with hydroxyapatite-embedded hyaluronic acid-alginate hydrogel for bone regeneration. (**A**) Schematic illustration of human umbilical cord mesenchymal stem cell-derived exosomes with hydroxyapatite-embedded hyaluronic acid-alginate hydrogel. (**B**) Transmission electron microscopy image of exosomes. (**C**) Size distribution of exosomes. (**D**) Western blot analysis of the exosome surface markers. (**E**) Release profiles of exosomes from the hydrogels with or without hydroxyapatite. * *p* < 0.05 compared to the control group; ^#^ *p* < 0.05 compared to the hydrogel group. (**F**) Confocal microscopy images of hydrogel with or without red fluorescence DiI-labeled exosomes. (**G**) Reconstructed 3D micro-CT images of the exosome-integrated hydrogels. Red circles indicate the defect area. (**H**) Quantitative analysis of bone regeneration using bone volume/tissue volume (BV/TV). Reproduced with permission from Yang et al. [164].

Approaches such as 3D culturing of donor cells, lentiviral modifications, miRNA overexpression or suppression, and hypoxic stimulation have been shown to significantly enhance exosome production, targeting, and osteogenic functions [10,51,185,189,190]. Fan et al. reported that exosomes derived from BMP antagonist, noggin-suppressed MSCs, were genetically modified with a lentiviral system to enhance the osteogenic properties of exosomes (Figure 2) [51]. These modified exosomes with a photocrosslinkable chitosan hydrogel demonstrated robust bone regeneration in a mouse nonhealing calvarial defect model. In addition, Deng et al. demonstrated hypoxia preconditioning of MSCs enhances the secretion of biglycan-rich exosomes (Hypo-exosomes), which significantly improve osteoblast activity, promoting proliferation, migration, and mineralization [163]. These effects are mediated through the activation of the PI3K/Akt pathway. To maximize therapeutic potential, a thermosensitive methoxy poly(ethylene glycol)-block-poly(l-alanine) hydrogel was developed to continuously release Hypo-exosomes over three weeks, significantly accelerating bone regeneration in animal models. This study highlights the potential of combining hypoxia-stimulated exosomes with hydrogel systems for effective bone tissue regeneration.

Furthermore, the biomaterials used in hydrogel scaffolds can influence cellular microenvironments and signaling, thereby modulating exosome release. The use of PEGMC in combination with β-TCP has demonstrated dual functionality in supporting both osteogenesis and angiogenesis [190,191]. Yang et al. developed an injectable hydrogel system with hydroxyapatite and hyaluronic acid-alginate to ensure long-term effectiveness (Figure 2) [164]. The composite hydrogels exhibited a slower and more controlled release of exosomes compared to pure hydrogels, releasing 71.20% (composite hydrogels) vs. 84.81% (pure hydrogels) of exosomes after 14 days. This slower release, combined with better mechanical strength, helps retain exosomes at the defect site for longer periods, enhancing their therapeutic effect. The combination of exosomes with this hydrogel significantly improved bone regeneration in rat cranial defect models.

In the area of alveolar bone defects, which are associated with an increased risk of periodontal diseases, exosome-integrated hydrogels combined with dental stem cells, such as dental pulp stem cells (DPSCs) and periodontal ligament stem cells (PDLSCs), have demonstrated effective regeneration in preclinical models. Zhang et al. investigated the osteogenic potential of exosomes derived from periodontal ligament stem cells (PDLSC-Exos) and their effect on repairing alveolar bone defects in rats [165]. PDLSCs were cultured, and their exosomes were purified and then tested for promoting bone marrow stem cell (BMSC) proliferation and osteogenic differentiation using CCK-8 and ALP staining. In vivo rats with alveolar bone defects were treated with alginate-gelatin crosslinked hydrogels containing PDLSC-Exos. Micro-CT and histological analyses revealed significant new bone formation in the exosome-loaded hydrogel group compared to control groups. The findings suggest that PDLSC-Exos delivered via hydrogel effectively enhance bone regeneration in alveolar defects. Furthermore, Isik et al. focused on using exosomes derived from human periodontal ligament fibroblasts (hPDLF-Exo) as a cell-free method to repair calvarial bone damage [166]. These exosomes were embedded into UV-responsive gelatin methacrylate (GelMA) hydrogels, creating a platform capable of regenerating critical-sized bone defects in middle-aged rats. In vitro results showed that human adipose mesenchymal stem cells cultured on GelMA/hPDLF-Exo hydrogels upregulated markers like RUNX2, ALP, and OSP, indicating enhanced osteogenic differentiation without additional growth factors. In vivo analyses, including micro-CT and histological methods, demonstrated increased bone mineralization in treated rats compared to controls. This suggests that the GelMA/hPDLF-Exo hydrogel can attract endogenous stem cells and promote bone healing.

Recent advancements in 3D printing technologies have enabled the precise construction of hydrogel scaffolds with complex spatial structures, offering customized solutions for bone defects [192,193]. These scaffolds create a conducive microenvironment for bone regeneration, providing mechanical support and promoting cellular activity [194,195,196]. Three-dimensionally printed double-network hydrogel scaffolds, comprising both a rigid and flexible layer, with MSC-derived exosomes have shown promising results in the regeneration of cartilage and subchondral bone. A first, rigid and brittle network is formed through cross-linking using GelMA, while a second, soft and ductile network is formed through Schiff base bonding. These hydrogels enhance bone marrow MSC attachment, migration, proliferation, and differentiation into both chondrogenic and osteogenic lineages. The bioactive exosomes provide sustained release, promoting tissue repair. In a rat osteochondral defect model, these 3D-printed bilayer scaffolds successfully regenerate both cartilage and subchondral bone. This 3D printing technology proposes a biomimetic approach and innovation in creating structured, functional, customizable scaffolds aimed at the targeted regeneration of complex bone tissues.

### 4.2. Fracture Healing

Fracture healing is often associated with significant pain and potential complications, especially in cases of open fractures where bone exposure increases the risk of infection, such as osteomyelitis [197,198]. Although various treatment strategies, such as fracture reduction, joint replacement, bone grafting, and bone stimulation, have been developed for nonunion fractures, the outcomes can still be inconsistent depending on the patient and the severity of the injury [199,200]. A critical aspect of the fracture healing process is the balance between osteoblast and osteoclast activity, as well as the ratio of M1 and M2 macrophages, both of which play a vital role in modulating the local inflammatory environment [201,202].

Recent advances in hydrogel-exosome systems for fracture repair have largely focused on immune regulation. Exosomes derived from genetically modified human umbilical vein endothelial cells (HUVECs), enriched with PD-L1, have been used to achieve immunosuppressive effects during the inflammatory phase of healing. The sustained release of PD-L1 via hydrogels has been shown to reduce the activity of CD8+ T cells, thus maintaining immune homeostasis essential for bone remodeling [167]. In addition, Chen et al. introduced an injectable, self-healing hydrogel composed of HA, SDF-1α, and M2 macrophage-derived exosomes (M2-Exos) [21]. The hydrogel exhibited excellent biocompatibility, antibacterial properties, hemostatic properties, and sustained release of bioactive agents, promoting the proliferation and migration of stem cells and endothelial cells, enhancing both osteogenesis and angiogenesis. The HA@SDF-1α/M2-Exos hydrogel accelerates fracture repair while addressing infection in vivo. A recent study explored the role of hydrogel-loaded exosomes derived from bone marrow mesenchymal stem cells in facilitating bone fracture repair [168]. The hydrogels were evaluated in vitro and found to recruit macrophages, reduce inflammation, promote angiogenesis, and support osteogenic differentiation. In a mouse fracture model, the hydrogels enhanced vascular migration and functional vascularization, promoting bone regeneration. Overall, exosome-loaded hydrogels accelerate fracture healing by modulating immune response and supporting bone tissue regeneration. In another approach, a cocktail therapy combining exosome-rich hyaluronic acid scaffolds with exosomes overexpressing miR-26a-5p and APY29 has demonstrated promising results [169]. This combination successfully suppressed pro-inflammatory cytokines while promoting M2 macrophage polarization and osteogenic differentiation, accelerating fracture healing.

Infection rates after open fractures, specifically fracture-related infections (FRIs), have been reported to range from 15% to 55% [203]. Treatment strategies typically include debridement, infection control, enhancement of bone healing, soft-tissue reconstruction, prevention of osteomyelitis, and functional recovery of the affected limb [204]. While surgical advancements have been made, effective treatment for nonunion still requires strong anti-inflammatory therapy and enhanced osteogenic differentiation. Yu et al. found that plasma exosomes from infected fracture nonunion patients (P-Exos) inhibited the osteogenic differentiation of bone marrow stromal cells, delaying fracture healing [170]. Overexpression of miR-708-5p in P-Exos was identified, which suppressed the Wnt/β-catenin pathway and hindered bone regeneration. Using a bacteria-sensitive hydrogel loaded with antagomiR-708-5p demonstrated effective antibacterial activity and promoted osteogenic differentiation, providing a promising therapeutic approach for infected fractures.

Angiogenesis plays a crucial role in the process of bone healing [205]. The formation of new blood vessels supplies oxygen and nutrients to the regenerating callus, which is highly metabolically active. Additionally, these vessels provide pathways for inflammatory cells, as well as cartilage and bone precursor cells, to access the site of injury. Umbilical cord mesenchymal stem cells (UC-MSCs) offer a promising cell source due to their ease of collection, robust proliferative and differentiation abilities, and minimal immunogenicity [206,207,208]. These characteristics have shown UC-MSCs to be effective in promoting bone regeneration, primarily through their capacity to enhance angiogenesis [209]. A study by Zhang and Hao et al. aimed to determine if exosomes from umbilical cord mesenchymal stem cells (UM-MSC-Exos) could enhance fracture healing primarily through angiogenesis [109]. Exosomes were isolated from UM-MSCs, characterized, and transplanted with a HyStem-HP hydrogel, comprising carboxymethyl hyaluronic acid-thiopropanoyl hydrazide with heparinthiopropanoyl hydrazide (HyStem-HP hydrogel, Catalog: GS315, Glycosan Biosystems, Salt Lake City, UT, USA), into a rat femoral fracture model. Bone healing progression and angiogenesis were assessed using radiographic and histological techniques. In vitro, uMSC-Exos promoted osteogenic and angiogenic gene expression, increased vascular endothelial growth factor (VEGF) and hypoxia-inducible factor-1α (HIF-1α) levels, and facilitated endothelial cell activities like migration and tube formation.

Despite these advances, much of the research has been limited to simple animal models. More complex fracture types, such as mandibular defects or subtalar osteoarthritis, still require further exploration in clinically relevant settings. Future studies on hydrogel-exosome systems for these intricate fracture models could provide more insight into their therapeutic potential.

### 4.3. Osteoarthritis

Osteoarthritis (OA) is a chronic and progressive condition that leads to the degradation of joint cartilage, formation of periarticular osteophytes, and local inflammation [210,211]. While often linked to aging, OA can also arise from joint injuries, genetic factors, or metabolic disorders [212]. The disease greatly affects patients’ quality of life, requiring long-term management and intervention to alleviate symptoms and slow progression [213].

Recent therapeutic approaches for OA have explored exosome-mediated drug delivery to target damaged bone and cartilage [213]. Wan et al. focused on developing new approaches to treat OA [171]. Although bone marrow mesenchymal stromal cell-derived exosomes show promise as drug carriers, their rapid clearance and low retention present challenges. To address these, researchers created a photocrosslinkable spherical hydrogel using GelMA and engineered exosomes modified with a WYRGRL (W) peptide for targeted cartilage affinity. This exosome–hydrogel-encapsulated system, named W-Exo@GelMA, was further loaded with LRRK2-IN-1, a small molecule inhibitor, and tested both in vitro and in vivo. Results showed that W-Exo-L@GelMA effectively targeted chondrocytes, reduced catabolic activity, and promoted anabolic processes in vitro. It also demonstrated strong anti-inflammatory effects and improved joint retention over 14 days, leading to enhanced cartilage repair in a mouse OA model. In addition, Cao et al. demonstrated the potential of exosomes derived from umbilical cord mesenchymal stem cells (UCMSC-EXOs) combined with targeted delivery and controlled release as a promising cell-free therapy for OA [172]. To enhance the effectiveness and retention of UCMSC-EXOs, they were modified with a chondrocyte-targeting polymer (chondrocyte affinity peptide-PEG-cholesterol) by integrating it into the lipid bilayer of exosomes and embedding in thiolated hyaluronic acid microgels, creating a dual-phase release system. Researchers also identified key miRNAs in UCMSC-EXOs, emphasizing the role of the p53 signaling pathway in their therapeutic effects. This approach significantly improved cartilage repair in a rat OA model.

**Figure 3:**
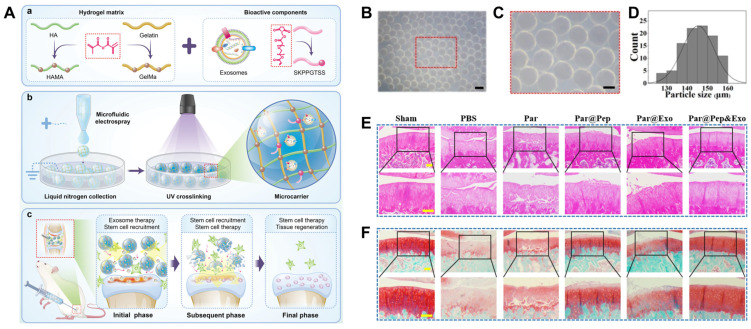
Exosome (Exo)-encapsulated stem cell-recruitment hydrogel microcarriers for osteoarthritis (OA) treatment. (**A**) Schematic illustration of Exo-encapsulated stem cell recruitment particles for OA treatment. (**a**) Hyaluronic acid (HA) and gelatin-based polymer matrix and bioactive components of hydrogel microcarriers. (**b**) Fabrication process of hydrogel microcarriers. (**c**) Application of microcarriers for OA treatment. (**B**–**D**) Characterization of particles and Exo. (**B**) Microscopic image of the particles. (**C**) The enlarged view of (**B**). (**D**) The size distribution of the particles. (**E**,**F**) H&E and Safranin O-fast green staining results. (**E**) H&E results after different particle treatments. (**F**) Safranin O-fast green staining results after the particle treatment. Par = microfluidic electrospray-generated hydrogel particles; Par@Pep = Par modified by SKPPGTSS peptides; Par@Exo = Par loaded with Exo; Par@Pep&Exo = Par@Pep loaded with Exo. Reproduced with permission from Yang et al. [173].

Another recent approach involved the development of exosome-encapsulated hydrogel microcarriers using a liquid nitrogen-assisted microfluidic electrospray technique (Figure 3) [173]. These microcarriers, formed through a precise droplet generation process and gentle cryogelation, exhibit consistent size distribution and high biocompatibility. The exosome-encapsulated hydrogel microcarriers include UV-crosslinked stem cell recruitment peptides (SKPPGTSS) with GelMA and methacrylated hyaluronic acid, enabling them to attract endogenous stem cells and sustain their release, which further supports cartilage repair through a combined action of stem cell recruitment and exosome release.

Recent studies highlight the role of the synovial lymphatic system (SLS) in OA progression, where impaired lymphatic drainage contributes to worsening symptoms [214,215]. Synovial macrophages influence lymphatic endothelial cells (LECs) within this system, with exosomes from macrophages potentially mediating this interaction [216]. A very recent study developed a thermosensitive injectable HA and Pluronic F-127 hydrogel system incorporating M2 macrophage-derived exosomes (M2Exo) for sustained release [174]. This M2Exo-loaded hydrogel enhances synovial lymphangiogenesis and improves lymphatic drainage in OA models, helping to slow disease progression and offering a new direction for OA treatment.

In another innovative approach, a biomimetic hydrogel inspired by mussel adhesion has been developed. Mussel-inspired hydrogels enable effective co-delivery of exosomes and icariin (ICA) for enhanced cartilage regeneration [175]. This system benefits from self-healing and adhesive properties, ensuring prolonged retention in the joint cavity. The synergistic effect of exosomes and ICA significantly improved cellular uptake, proliferation, and migration while reducing cartilage degradation markers. In osteoarthritis models, the hydrogel system based on chitosan, catechol-modified chitosan, β-glycerophosphate disodium salt hydrate, and dialdehyde-functionalized polyethylene glycol demonstrated improved cartilage protection and structure preservation.

Additionally, gene-editing strategies using hydrogels have emerged. The abnormal suppression of FGF signaling is a key factor in OA and joint disorders, but using FGF18-based therapies faces challenges like short stability and the need for frequent dosing [217,218]. Recently, Chen et al. introduced a CRISPR/Cas9-based strategy to activate the FGF18 gene directly in OA chondrocytes, utilizing hybrid exosomes (CAP/FGF18-hyEXO) with chondrocyte-affinity peptides for precise targeting [176]. These exosomes are further encapsulated into hyaluronic acid-based hydrogel microspheres, forming an injectable system that offers sustained hydration and lubrication. This exosome–hydrogel system effectively enhanced cartilage repair, reduced inflammation, and prevented extracellular matrix breakdown in vitro and in vivo.

### 4.4. Others

Intervertebral disc degeneration (IVDD) is a common condition associated with chronic pain and limited mobility [219,220]. It occurs due to the degeneration of nucleus pulposus cells (NPCs), leading to the loss of disc function and structure [221]. To address this, hydrogel-exosome systems have emerged as a promising non-invasive treatment approach. In one study, exosomes derived from chondrocyte endplate stem cells (CESCs), engineered to overexpress Sphk2, were combined with costal cartilage extracellular matrix (ECM) hydrogels for targeted injection [177]. When injected near the cartilage endplate in rats, the ECM-Gels release Sphk2-engineered exosomes (Lenti-Sphk2-Exos). These exosomes deliver Sphk2 to nucleus pulposus cells (NPCs), activating the PI3K/p-AKT pathway and promoting autophagy. This method effectively reduces IVDD progression in vivo. Another innovative strategy involved exosomes enriched with GLRX3 (Exos-GLRX3) through hypoxic preconditioning, which improved the antioxidant defenses of nucleus pulposus cells, reducing reactive oxygen species (ROS) and slowing cellular aging [178]. When combined with a ROS-responsive hydrogel based on dopamine-functionalized gelatin and borax-coupled aldehyde-modified chondroitin sulfate, the Exos-GLRX3 showed promise in reducing disc degeneration by restoring ECM balance and protecting mitochondrial function in a rat model. These hydrogel-exosome systems provide a novel therapeutic option by addressing the cellular and molecular mechanisms underlying IVDD, offering potential relief from the associated pain and mobility issues.

Anterior cruciate ligament (ACL) reconstruction faces challenges in achieving effective grafted tendon-bone integration [222,223]. Exosomes derived from hypoxia-preconditioned bone marrow mesenchymal stem cells (Hypo-Exos) show promise in enhancing angiogenesis and osteogenesis, aiding this integration process [179]. Hypo-Exos significantly improved endothelial cell proliferation, migration, and tube formation in vitro and promoted better bone tunnel healing in animal models compared to normoxia-derived exosomes. These findings suggest that Hypo-Exos can accelerate graft-bone integration and improve the structural and biomechanical properties of the peri-graft bone after ACL reconstruction.

Growth plate cartilage has a limited ability to repair itself, often resulting in improper bone bridge formation and growth defects in children after an injury [224,225]. Current surgical interventions are invasive and do not consistently yield reliable outcomes. After an injury, the loss of ECM and the impact of inflammation on cartilage matrix proteins impede the regeneration of chondrocytes. To address this, a hydrogel mimicking the ECM was developed, loaded with exosomes from bone marrow mesenchymal stem cells (BMSCs) and enhanced with aldehyde-functionalized chondroitin sulfate within a gelatin methacryloyl structure [180]. This exosome–hydrogel composite enhanced ECM production, reduced inflammation, and supported chondrocyte anabolism, ultimately aiding in growth plate repair through effective ECM remodeling in vitro and in vivo.

## 5. Future Perspectives and Conclusions

Despite the significant advancements in exosome-integrated hydrogels for bone tissue engineering, several challenges remain in translating these materials into clinical practice. A primary challenge lies in the scalability and consistent production of exosome-integrated hydrogels. The isolation of exosomes is complex and labor-intensive, often resulting in variable yields. Ensuring reproducibility and scalability without compromising bioactivity is essential. Developing standardized protocols and advanced biomanufacturing, like automated bioreactor systems, could overcome these issues.

The integration of exosomes into hydrogels also presents technical challenges. Uniform dispersion of exosomes within the hydrogel matrix is crucial for consistent therapeutic effects; poor dispersion can lead to uneven release profiles and local agglomeration. Techniques such as microfluidic mixing or surface modification of exosomes may help achieve even distribution.

Controlled release of exosomes is another challenge. Hydrogels offer a promising platform for localized delivery, but optimizing release kinetics to match the healing process is complex. Hydrogel degradation rate, crosslinking density, and interactions between exosomes and the hydrogel network influence release profiles. Further research should refine these parameters to maximize regenerative potential.

Biocompatibility and immunogenicity are critical concerns. While promising in preclinical studies, long-term evaluations are necessary to confirm that these materials do not elicit immune responses or other adverse effects. Both exosomes and hydrogel matrices need thorough biocompatibility assessments, especially for chronic use. Furthermore, immune responses based on the exosome source (autologous or allogeneic) require careful consideration to ensure therapeutic efficacy without adverse effects in clinical applications.

Regulatory challenges also exist for exosome-integrated hydrogels. These materials need to meet rigorous safety and efficacy standards from regulatory bodies such as the U.S. Food and Drug Administration (FDA) and the European Medicines Agency (EMA). As a novel combination of biological and synthetic components, they require unique evaluation protocols. Developing standardized testing for quality and performance will be critical. Exosomes, as biologically active vesicles, necessitate comprehensive safety assessments to ensure long-term efficacy and control possible complications. Additionally, the costs associated with exosome production, isolation, and integration into hydrogels are substantial, posing a barrier to large-scale manufacturing and clinical application. Implementing cost-effective production methods and scalable technologies will be essential to make exosome-integrated hydrogels a viable option for widespread clinical use.

Future research should prioritize new strategies to enhance stability, bioactivity, and controlled release of exosomes. “Smart” hydrogels that respond to physiological cues, like pH or enzyme activity, could offer more dynamic, adaptable treatment options and improve bone tissue engineering outcomes. Additionally, combining exosome-based therapies with emerging technologies like 3D bioprinting and gene editing presents exciting possibilities. Exosome-integrated hydrogels combined with 3D bioprinting could precisely fabricate scaffolds mimicking natural bone structure, enhancing integration and function. Gene editing tools, such as CRISPR/Cas9, could engineer donor cells to produce exosomes with specific therapeutic properties.

Furthermore, we conducted a search on the ClinicalTrials.gov website on 20 November 2024, using the keywords “exosome”, “hydrogel”, and “bone”. This search did not yield any results. Currently, no clinical trials have been initiated or completed for exosome-integrated hydrogels specifically targeting bone diseases or injuries. However, approximately ten clinical trials were identified that involve the use of exosomes or extracellular vesicles for certain bone diseases and injuries. At this stage, significant challenges remain in confirming the efficacy and safety of exosome-integrated hydrogels for clinical applications, particularly in the context of bone regeneration and repair.

Interdisciplinary collaboration between researchers, clinicians, and regulatory bodies is essential to navigate the path to clinical translation. This collaboration will ensure that exosome-integrated hydrogels meet the highest safety and efficacy standards, leading to effective treatments for bone defects and other regenerative needs.

In conclusion, while exosome-integrated hydrogels hold great promise for bone tissue engineering, significant challenges remain. Overcoming these through research and innovation is crucial for realizing the technology’s full potential and advancing its clinical applications. As regenerative medicine evolves, exosome-integrated hydrogels are poised to contribute significantly to next-generation therapies, enhancing function and quality of life for patients with bone injuries and diseases.

## Figures and Tables

**Table 1:** Criteria for successful exosome extraction or isolation techniques.

	Technique	Differential Centrifugation	Density Gradient Centrifugation	Ultrafiltration	Size Exclusion Chromatography	Affinity Nanoparticle-Based Isolation	Polymer Precipitation	Microfluidic Technologies	Commercial Kits
Criteria ^a^									
Complexity		2	2	3	1	2	2	3	3
Yield		3	3	3	3	3	1	3	3
Cost		2	2	2	2	1	1	1	1
Purity		2	3	2	3	3	2	3	3
Processing time		1	1	3	1	1	2	1	3
Special equipment		1	1	1	1	2	3	1	2
Scale-up		2	1	3	1	1	3	1	1
Professional skills for operation		2	2	2	1	1	3	1	3

^a^ 1 and 3 are the worst (lowest) and the best (highest) score for criteria, respectively.

**Table 3:** Recent applications of exosome-integrated hydrogels in bone tissue engineering.

Cell Source/Preparation Method of Exosomes	Materials for Hydrogel	Active Agent	Key Findings	Ref.
M2 macrophages/ Ultracentrifugation	Hyaluronic acid	SDF-1α	Stimulated extracellular matrix mineralization and HUVEC tube formation. Enhanced osteogenesis and angiogenesis both in vivo and in vitro	[21]
Human mesenchymal stem cells/ Gradient ultracentrifugation	Glycidyl methacrylate, glycol chitosan	miRNA-29a	Enhanced osteogenesis by EMs through noggin suppression via inhibition of miR-29a. Promoted calvarial bone repair in vivo.	[51]
Human umbilical cord mesenchymal stem cells/ Ultrafiltration	Thiol-modified hyaluronan, HA, and thiol-modified heparin	None	Increased the expression of vascular endothelial growth factor (VEGF) and hypoxia inducible factor-1α (HIF-1α). Enhanced HUVECs proliferation, migration and tube formation. Enhanced pro-angiogenesis and fracture repair.	[109]
Lipopolysaccharide (LPS)-trained bone marrow-derived MSCs/Differential ultracentrifugation	Thixotropic injectable silk fibroin	Lapnoite	Enhanced the regenerative effect of BMSC-EVs. Enhanced the immunoregulatory effect of EVs by promoting the M1–M2 transition of macrophages and increasing EV secretion.	[162]
Bone marrow MSCs/Gradient ultracentrifugations	Poly(ethylene glycol)/polypeptide copolymers	biglycan	Accelerated bone regeneration in 5 mm rat cranial defects. Promoted osteoblast differentiation and mineralization.	[163]
Human umbilical cord mesenchymal stem cells/Ultracentrifugation	Hydroxyapatite (HAP)-embedded in situ cross-linked hyaluronic acid-alginate (HA-ALG)	None	Effectively promote the proliferation, migration, and osteogenic differentiation of a murine calvariae preosteoblast cell line in vitro.. Enhanced bone regeneration in vivo.	[164]
Periodontal ligament stem cells/Ultracentrifugation	Alginate-gelatin crosslinked	None	The hydrogel successfully delivered periodontal ligament stem cells derived exosome. Repaired alveolar bone defects in rats in vivo.	[165]
Human periodontal ligament fibroblasts/Ultracentrifugation	UV-responsive gelatine methacrylate	None	Growth factors-free induction of osteogenic differentiation in hMSCs. Higher new bone mineralization that providing bone damage repair.	[166]
Human Umbilical Vein Endothelial Cell/Ultracentrifugation	Hydrazide-grafted gelatin	PD-L1	Inhibited T cell activation in peripheral lymphatic tissues. Enhancement of bone repair and regeneration.	[167]
Bone marrow mesenchymal stem cells/Ultracentrifugation	Glycol chitosan, silk fibroin, chondroitin-6-sulfate, dialysis-purified maleic anhydride-modified polyethylene glycol	None	Facilitated vascular migration. Promoted the formation of large vessels. Enabled functional vascularization during bone repair. Promote fracture healing and angiogenesis in vivo.	[168]
Endothelial cells/Sequential centrifugation	Hyaluronic acid	APY29	Promoted osteogenic differentiation and osteoclast differentiation. Enhanced therapeutic effect in a fracture model.	[169]
Infected fracture nonunion patients/Ultracentrifugation	Hyaluronic acid	microRNA-708-5p	Accelerated fracture healing via promotion of osteoblast differentiation and anti-inflammation.	[170]
Bone marrow mesenchymal stromal cell/ Ultracentrifugation	Gelatin methacryloyl	LRRK2-IN-1	Inhibited OA-related inflammation and immune gene expression. Enhanced cartilage repair ability in the OA murine model.	[171]
Umbilical cord-derived mesenchymal stem cells/ Gradient centrifugation	Hyaluronic acid	Chondrocyte-targeting peptide	Promoted cartilage matrix synthesis of OA chondrocytes. Sustained release extended the retention of exosomes in vivo.	[172]
Human umbilical cord mesenchymal stem cells/ CytoNiche 3D FloTrix^®^ Process	Hyaluronic acid methacryloyl, gelatin methacryloyl	Acryloylated stem cell recruitment peptides SKPPGTSS	Recruited endogenous stem cells to promote cartilage repair. Enhanced the therapeutic performance through synergistic effects.	[173]
M2 macrophages/ Sequential centrifugation	Hyaluronic acid	None	Exhibited a controlled release profile of exosomes that efficaciously fostered synovial lymphangiogenesis	[174]
Bone marrow mesenchymal stromal cell/ Exosome Isolation Kit	Chitosan, β-glycerophosphate disodium salt hydrate, dialdehyde-functionalized polyethylene glycol	Icariin	Facilitated the cell attachment and migration. Promoted the cartilage regeneration.	[175]
Cas9-CAP/HEK293 cells/ Gradient centrifugation	Hyaluronic acid	CAP plasmid, Cas9 mRNA	Promoted cartilage regeneration, decreased inflammation, prevented ECM degradation both in vitro and in vivo	[176]
Cartilage endplate stem cells /Ultracentrifugation	Costal cartilage extracellular matrix	Sphk2	Inhibited disc degeneration through PI3K/AKT signaling both in vitro and in vivo	[177]
Bone marrow mesenchymal stromal cell/Differential ultracentrifugation	Dopamine-functionalized gelatin	GLRX3	Exhibited high efficiency in attenuating mitochondrial damage, decreasing local senescence state, and restoring the matrix deposition of NP cells	[178]
Bone marrow mesenchymal stromal cell/Differential ultracentrifugation	GelMA, HA-NB, and photo-initiator	None	Enhanced peri-graft bone formation and remodeling that enhances the grafted tendon-bone tunnel integration	[179]
Bone marrow mesenchymal stromal cell/Ultracentrifugation	Aldehyde-functionalized chondroitin sulfate, gelatin methacryloyl	None	Promoted cartilage regeneration and reduced the inflammatory response	[180]

## Data Availability

No new data were created or analyzed in this study.
